# Enhanced Gamma Activity and Cross-Frequency Interaction of Resting-State Electroencephalographic Oscillations in Patients with Alzheimer’s Disease

**DOI:** 10.3389/fnagi.2017.00243

**Published:** 2017-07-26

**Authors:** Jing Wang, Yuxing Fang, Xiao Wang, Huichao Yang, Xin Yu, Huali Wang

**Affiliations:** ^1^Peking University Sixth Hospital (Institute of Mental Health) Beijing, China; ^2^National Clinical Research Center for Mental Disorders, Key Laboratory of Mental Health, Ministry of Health, Peking University Beijing, China; ^3^Beijing Municipal Key Laboratory for Translational Research on Diagnosis and Treatment of Dementia Beijing, China; ^4^National Key Laboratory of Cognitive Neuroscience and Learning, Beijing Normal University Beijing, China; ^5^IDG/McGovern Institute for Brain Research, Beijing Normal University Beijing, China

**Keywords:** Alzheimer’s disease, oscillations, resting-state, EEG, cross-frequency coupling

## Abstract

Cognitive impairment, functional decline and behavioral symptoms that characterize Alzheimer’s disease (AD) are associated with perturbations of the neuronal network. The typical electroencephalographic (EEG) features in AD patients are increased delta or theta rhythm and decreased alpha or beta rhythm activities. However, considering the role of cross-frequency couplings in cognition, the alternation of cross-frequency couplings in AD patients is still obscure. This study aims to explore the interaction dynamics between different EEG oscillations in AD patients. We recorded the resting eye-closed EEG signals in 8 AD patients and 12 healthy volunteers. By analyzing the wavelet power spectrum and bicoherence of EEG, we found enhanced gamma rhythm power in AD patients in addition to the increased delta and decreased alpha power. Furthermore, an enhancement of the cross-frequency coupling strength between the beta/gamma and low-frequency bands was observed in AD patients compared to healthy controls (HCs). We propose that the pathological increase of ongoing gamma-band power might result from the disruption of the GABAergic interneuron network in AD patients. Furthermore, the cross-frequency overcouplings, which reflect the enhanced synchronization, might indicate the attenuated complexity of the neuronal network, and AD patients have to use more neural resources to maintain the resting brain state. Overall, our findings provide new evidence of the disturbance of the brain oscillation network in AD and further deepen our understanding of the central mechanisms of AD.

## Introduction

Alzheimer’s disease (AD) is a neurodegenerative disorder characterized by cognitive deficits, disorders of activities of daily living and behavioral disturbance, with widespread cortical atrophy mainly localized in the temporal-parietal lobe (Marceglia et al., 2016).

Electroencephalography (EEG) rhythmical oscillations in the low (delta, theta and alpha) and high (beta and gamma) frequencies have been demonstrated to be linked to a broad variety of perceptual, sensorimotor and cognitive processes (Schroeder and Lakatos, 2009). Increasing evidence has shown that the resting state EEG rhythms may reveal abnormalities of the basic neurophysiological mechanisms that underlie vigilance and cognition in AD subjects. These abnormal EEG rhythms are thought to be associated with functional cortical disconnections, resulting in the death of cortical neurons, axonal pathology and neurotransmission deficits (Tsolaki et al., 2014).

Previous studies have shown that compared to normal elderly subjects, AD patients are characterized by high power of delta (<4 Hz) and/or theta (4–7 Hz) rhythms and low power of alpha (8–12 Hz) and/or beta (13–30 Hz) rhythms (Babiloni et al., 2016). In addition, other studies reported the increased delta coherence, decreased theta and alpha coherence, higher alpha and lower delta and beta small world characteristics of connectivity (Marceglia et al., 2016; Vecchio et al., 2017) in AD patients. Some reports (Tsolaki et al., 2014) also found the correlation between the degree of the EEG abnormality and the cognitive impairment in AD patients, including the correlation between EEG delta, theta, and alpha activities and Mini Mental Status Examination (MMSE) scores, between theta/gamma ratio and worse performance in nonverbal learning tests, between lagged phase synchronization in the theta band and MMSE scores.

However, the alteration of gamma band during resting state in AD patients is still obscure. Gamma rhythm has been verified to play an important functional role during cognitive functions (Missonnier et al., 2010). Some studies have showed disturbed task-induced gamma dynamics in AD patients and mouse models of AD, including increased auditory steady state gamma amplitudes (Osipova et al., 2006), delayed gamma responses, higher gamma fractal dimension values (Basar et al., 2016), increased high-frequency coherence (Hogan et al., 2003), and decreased high-frequency spectral powers in the frontal and temporal areas (Koberda et al., 2013; Stoiljkovic et al., 2016). Therefore, it is also interesting for exploration the abnormality of gamma rhythm in AD patients.

Furthermore, different EEG oscillations in the frequency domain are not independent; in contrast, the mutual interaction of cross-frequency oscillations regulate multi-network integration (Buzsáki and Watson, 2012). For example, the magnitude of theta-gamma coupling in the hippocampal region varied with working memory load (Axmacher et al., 2010). One recent study found that impaired theta-gamma phase-amplitude coupling was associated with the cognitive deficits in the mice of the AD model (Zhang et al., 2016). However, the abnormality of cross-frequency couplings in AD patients is still obscure, and it is also critical for understanding the underlying mechanisms of the multi-dimensional decline in AD patients.

Considering the role of brain oscillations and cross-frequency interactions in memory and cognition, in this study, we aimed to explore the interaction dynamics between different EEG oscillations, including the power information and cross-frequency coupling in AD patients compared with the healthy volunteers.

## Materials and Methods

### Participants

This study was approved by the ethics committee of the Peking University Institute of Mental Health. Written informed consent was obtained from the participants and their families at the beginning of the study. No vulnerable populations were involved in this study. All subjects were Chinese Han, right-handed and more than 55 years old. Patients with AD were prospectively recruited from the Dementia Care and Research Center, Peking University Institute of Mental Health. A clinical diagnosis of AD was made according to the criteria for dementia cited in the International Classification of Diseases, 10th Revision (ICD-10; World Health Organization, 1999) and the criteria for probable AD of the National Institute of Neurological and Communicative Disorders and the Stroke/AD and Related Disorders Association (NINCDS-ADRDA; McKhann et al., 1984). Other inclusion criteria were as follows: more than 6 months’ duration of the disease and MMSE score of 15–24. Exclusion criteria comprised any type of evidence of other forms or causes of dementia, such as frontotemporal dementia, vascular dementia, Parkinson’s disease, Lewy body dementia, metabolic syndrome, nutritional deficits and tumors. The healthy elderly subjects had no history of neurological or major psychiatric disorder. They underwent medical, neurological and psychiatric assessments, including MMSE and clinical dementia rating (CDR), to exclude actual neurocognitive disorders and major psychiatric symptoms (including abuse of substances). Finally, this study involved 8 patients with AD and 12 healthy elderly individuals, which were carefully matched for age, gender and years of education.

### Neuropsychological Assessment

Each participant underwent a thorough neuropsychological assessment, including the MMSE, immediate and delayed memory test of Wechsler memory scale (WMS), Raven’s combined progressive matrices (RCPM), Beck depression inventory (BDI-II), digit span test, and attentional matrices test. The function was assessed with the activities of daily living scale (ADL).

### EEG Recording and Pre-Processing

We employed the 32-channel EEG system (bandpass: 0.01–100 Hz; Brain Products GmbH, Munich, Germany) for the collection of EEG signals. The 32 scalp electrodes were positioned over the whole head according to the 10–20 System, with the Cz electrode as the reference electrode. Electrode impedance was kept below 20 kΩ. All subjects were kindly asked to stay relaxed with their eyes closed and not to move or talk; then, 5 min of EEG signals were recorded.

Signals were analyzed offline with Matlab (The Mathworks, Natick, MA, USA) EEGLAB software. All recorded artifact-free EEG data were re-referenced off-line to a common average. The EEG signals were digitized at a sampling rate of 500 Hz, re-referenced to an average of residual channels, band-pass filtered in the range of 1–45 Hz to avoid the interference of 50 Hz signals and subsequently inspected for artifact rejection; conspicuous baseline drift and artifacts caused by eye movement were eliminated by visual inspection on time series, and the automatic artifact rejection threshold was set to ±100 μV.

### Data Analysis

We used the wavelet power spectrum analysis method to obtain the power of the spontaneous EEG activities (Shaw et al., 1999). The Morlet wavelet transform was employed with the wavelet central angle frequency of 6 (*ω* = 6). The following five frequency bands were examined: 1–4, 4–8, 8–13, 13–30, 30–45 Hz, corresponding to delta, theta, alpha, beta and gamma bands, respectively (Mantini et al., 2007), with a step of 0.5 Hz.

Cross-frequency interaction often reflects the synchronization of networks, and the power spectrum could not include the phase information. To evaluate the degree of cross-frequency phase couplings, we measured the co-modulation of oscillations between two frequency bands with general harmonic wavelet bicoherence (Li et al., 2009). The bicoherence method is the normalized form of the bispectral analysis, which we described in our previous study (Li et al., 2009; Wang et al., 2013). Briefly, signals were divided into a series of 2-s epochs, with an overlap of 75%. Then, for each epoch, bicoherence values were computed in all pairs of frequencies from 1 Hz to 45 Hz, with a step of 1 Hz and a bandwidth of 2 Hz. Finally, the filtered wavelet bicoherence value (abbreviated as FIWBIC) was calculated using the same epoch as the power analysis above.

For the demographic information, an unpaired *t*-test was used to compare the difference between two groups.

For power spectral data, a nonparametric permutation test (Wang et al., 2015) was used to identify the difference in the frequency ranges of EEG power between the two groups (the number of permutations = 10,000, *P* < 0.05). The False Discorvery Rate (FDR; Benjamini and Yekutieli, 2001) was conducted for multiple comparisons correction (the number of multiple comparisons = 5).

For the wavelet bicoherence data, we focused on the characteristics to determine the differences between two groups; prior to the contrast, the characteristic total bicoherence value at the frequency bands (${\text{f}}_{\text{j}}^{\text{L}}\leq {\text{f}}_{\text{j}}\leq {\text{f}}_{\text{j}}^{\text{U}}$ and ${\text{f}}_{\text{k}}^{\text{L}}\leq {\text{f}}_{\text{k}}\leq {\text{f}}_{\text{k}}^{\text{U}}$) was extracted, which was defined as $b=\sum \sum b_{\text{xxx}}^{2}(f_{\text{j}},f_{\text{k}})$, where *b*_xxx_ is FIWBIC. This value reflects a measure of the degree of quadratic phase coupling (QPC) between frequency bands and can be used to measure the phase coupling strength between different oscillations (Li et al., 2009). Then, a nonparametric permutation test was employed to determine marked differences (the number of permutations = 10,000), with a *P* value less than 0.05 (FDR corrected, the number of multiple comparisons = 10) as a statistically significant standard.

## Results

There was no significant difference in age, gender, education level, intelligence and BDI-II score between the AD and healthy control (HC) groups. As expected, a remarkable difference was found for the MMSE score (*P* < 0.0001), activities of daily living (*P* = 0.0021), WMS immediate and delayed memory score (*P* < 0.0001), digit span backward score (*P* = 0.0157), percentage of correctness (*P* = 0.0038) and efficiency (*P* = 0.0052) for attentional matrices test in AD patients compared with the HC group (Table 1).

**Table 1:** Demographics and clinical characteristics of the participants.

	AD (*N* = 8)	HC (*N* = 12)	*P*
Gender (men/women)	4/4	7/5	0.7136
Age	76.88 ± 0.74	73.67 ± 2.30	0.2834
Years of education	13.13 ± 0.91	14.68 ± 0.67	0.2041
MMSE	22.5 ± 1.35	29.08 ± 0.29	<0.0001***
BDI-II	8 ± 1.78	4.92 ± 1.15	0.1445
WMS			
Immediate memory	2.75 ± 0.98	9.46 ± 0.74	<0.0001
Delayed memory	1.38 ± 1.02	12.73 ± 0.99	<0.0001
RCPM	25.88 ± 1.06	30.27 ± 1.83	0.0774
ADL	30.75 ± 3.36	20.36 ± 0.36	0.0021**
Digit span			
Forward	6.63 ± 0.32	7 ± 0.21	0.3292
Backward	3.25 ± 0.31	5 ± 0.50	0.0157*
Attentional matrices			
PC (%)	84.59 ± 2.40	94.09 ± 1.68	0.0038**
PE (%)	67.86 ± 6.74	88.16 ± 2.31	0.0052**

*P < 0.05, **P < 0.01, ***P < 0.001.

**Figure 1:**
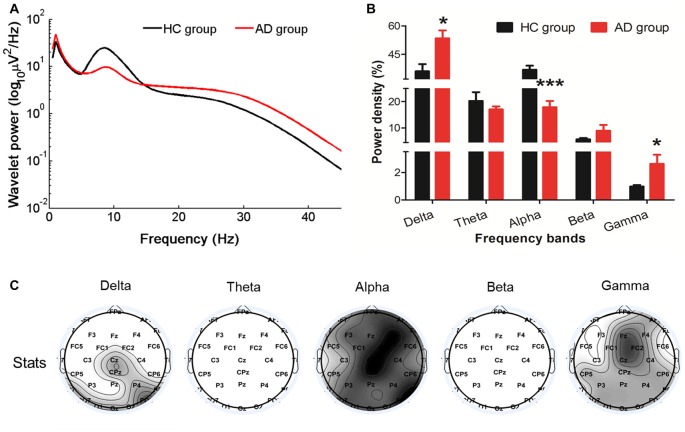
Changes in the EEG power spectra in the Alzheimer’s disease (AD) group compared with the healthy control (HC) group. **(A)** The average absolute EEG power in HCs (black line) and AD patients (red line). The *Y-axis* represented the power value and the *X-axis* represented frequency bands. **(B)** The relative power (normalized to the overall power) in five frequency bands. A marked increase of the relative power in delta and gamma frequencies and a decrease in alpha frequency was observed in the AD group (red columns) compared with the power in the HC group (black columns). *P_FDR_ < 0.05, ***P_FDR_ < 0.001 compared with the HC group. All data were expressed as the means ± SEM (*n* = 8–12). **(C)** Topographic distribution of statistical significances in EEG power in the AD group compared with the HC group. Values were color coded. Black: *P* < 0.001; dark gray: *P* < 0.01; light gray: *P* < 0.05; white: not significant, uncorrected for the number of electrodes tested.

Compared with the HC, the relative resting-state wavelet power of delta (*P* = 0.0149) and gamma (*P* = 0.0194) frequency oscillations increased, and the power of alpha bands decreased (*P* = 0.0003) in the AD group (Figures 1A,B). Furthermore, we found that the increased delta was mainly located in the central-parietal and occipital areas, the decreased alpha was widespread around the entire cortical area, and the increased gamma was mainly located in the midline frontal, central-parietal and occipital areas (Figure 1C).

**Figure 2:**
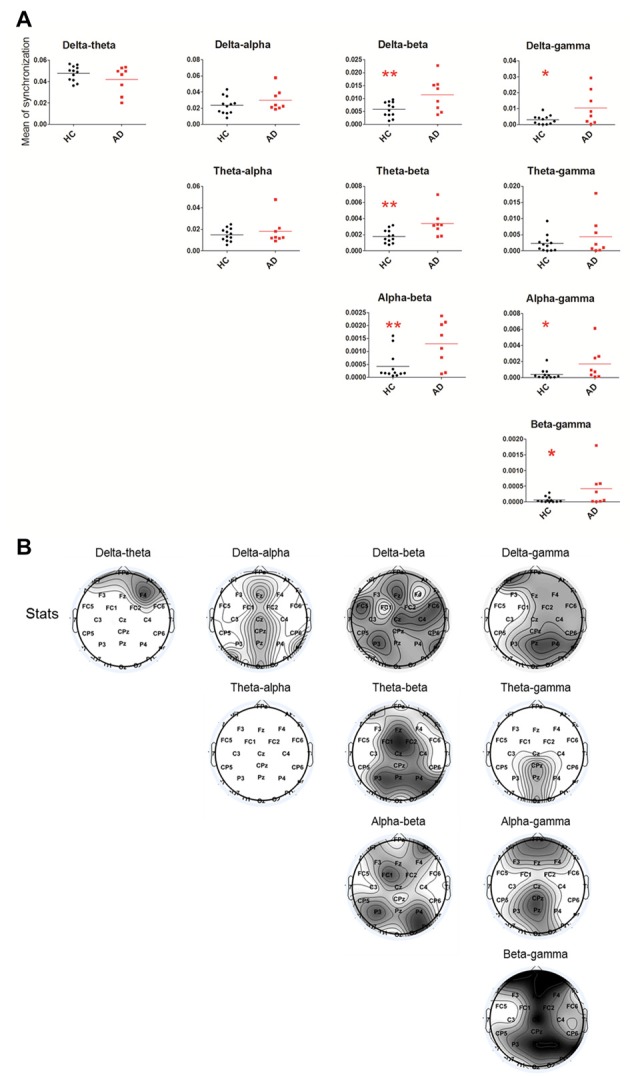
Statistical analysis of filtered wavelet bicoherence value (FIWBIC) in the AD group compared with the HC group and topgraphic distribution of statistical differences. **(A)** Scatter plot for phase coupling at local frequency bands in the HC group (black) and AD group (red).The *Y-axis* represented the mean of synchronization. The synchronization values increased between between beta and delta/theta/alpha, and between gamma and delta/alpha/beta. *P_FDR_ < 0.05, **P_FDR_ < 0.01, (*n* = 8–12). **(B)** Topographic distribution of statistical significances in cross-frequency coupling between two groups. Values were color coded. Black: *P* < 0.001; dark gray: *P* < 0.01; light gray: *P* < 0.05; white: not significant, uncorrected for the number of electrodes tested.

Relative to the HC group, the cross-frequency coupling strength between beta and delta (*P* = 0.0098), beta and theta (*P* = 0.0035), beta and alpha (*P* = 0.0079), gamma and delta (*P* = 0.0184), gamma and alpha (*P* = 0.0079) and gamma and beta (*P* = 0.0162) in the AD group (Figure 2A) was significantly increased, indicating stronger synchronization among the high-low frequency bands. Furthermore, we found that the increased delta-theta coupling was mainly located in prefrontal, the increased delta-beta coupling was widespread around the entire cortical area, the increased delta/beta and gamma couplings were located in cortical areas except for left temporal area, the theta-gamma coupling was increased in midline parietal-occipital area, whereas the increased theta/alpha-beta couplings and alpha-gamma coupling were located in cortical areas except for bilateral temporal lobes in AD group (Figure 2B).

However, we did not observe a significant relationship between the wavelet power, phase coupling strength with the neurocognitive performance (*P* > 0.05).

## Discussion

By analyzing the wavelet power spectrum and bicoherence of EEG, we found that, in AD patients, expected for the increased delta and decreased alpha power, the resting-state gamma rhythm power was enhanced and was mainly located in the midline frontal, central-parietal and occipital areas. Furthermore, the enhancement of the cross-frequency coupling strength between beta and delta/theta/alpha, between gamma and delta/alpha/beta were observed in AD patients compared to HCs, and were also mainly located in the frontal, parietal-occipital areas.

In our study, compared to HCs, we found more pronounced delta power mainly located in the central-parietal and occipital areas and less prominent widespread alpha power in AD patients. In line with previous studies, these changes were recognized as typical EEG alterations in patients with dementia (Babiloni et al., 2010, 2013). Notably, alpha oscillations have been suggested to play an important role in cognitive and memory processing (Klimesch, 1999). Therefore, the increase of delta and weakened alpha rhythm in our study may reflect deficits in brain activity or cognitive decline (Hsiao et al., 2013).

Interestingly, we found AD patients showed higher power in resting-state high-frequency oscillation, especially in gamma band, which mainly located in the midline frontal, central-parietal and occipital areas. It was inconsistent with the findings in some report (Koberda et al., 2013; Stoiljkovic et al., 2016), in that article, decreased stimulation-elicited hippocampal gamma power was reported in mice of AD model. This divergence might be related to different states of EEG recording and the severity of the disease. Gamma rhythm has been indicated to play a relevant functional role during perceptual, executive and mnemonic processes (Missonnier et al., 2010). It arises from the inhibitory GABAergic parvalbumin-expressing (PV) interneurons network (Buzsáki and Wang, 2012). Many mouse models of AD have been generated with a growing realization that dysfunction of the GABAergic network plays a role in AD pathogenesis (Verret et al., 2012; Hazra et al., 2013; Ma and McLaurin, 2014). Therefore, it is reasonable that abnormal on-going gamma oscillation exists in AD patients.

In view of the relationship between gamma oscillation and neurotransmitters, the GABAergic interneurons receive excitatory inputs through N-methyl-D-aspartate (NMDA) receptors and are inhibited by the activation of metabotropic muscarinic receptors (mAChRs) on its terminal (Picciotto et al., 2012); therefore, dysfunction of acetylcholine (Ach) and NMDA neurotransmission may result in abnormalities of gamma oscillation. Memory and cognitive decline in aging and dementia are associated with synaptic/extrasynaptic NMDAR over-activation by glutamate and decreased cholinergic function. Additionally, Aβ deposition, which played an important role in pathophysiology of AD, interfered with NMDA neurotransmission and suppressed NMDAR-dependent synaptic functions (Cissé et al., 2011). Based on these data, it is conceivable that the enhanced power in gamma oscillations in our results might reflect the continuous gamma discharge resulting from the over-activation of the GABAergic interneuron network induced by extrasynaptic NMDAR over-activation and decreased cholinergic function in AD patients.

Considering the integration of cross-frequency interactions in different networks, we further found an increase in the cross-frequency coupling strength between the high-frequency bands (beta/gamma) and low-frequency bands (delta/theta/alpha), which were mainly located in frontal, parietal-occipital areas. It has been accepted that low frequency might be involved in the integration across widely spatially large scale networks and high frequency (beta and gamma) oscillations are distributed over a more limited topographic area tuning the local scale network interactions. The coupling between slow and high oscillations has been observed in various regions during rest, the processing of visual and auditory stimuli, memory operations, and working memory maintenance (Palva and Palva, 2007; Roux et al., 2013; Lega et al., 2016; Park et al., 2016). These findings suggest that the slow-fast rhythm interactions play an important role in coordinating information processing. Cross-frequency phase synchrony between alpha, beta and gamma oscillations has been thought to coordinate the selection and maintenance of neuronal object representations during perception, consciousness and working memory (Palva and Palva, 2007). The existence of a beta-delta interaction had been associated with anxiety, a prevalent neuropsychiatric symptom observed in AD patients (Miskovic et al., 2010). Reduced theta/beta ratio may reflect lower reward-gain motivation and higher anxiety levels (Putman et al., 2010). Therefore, in our study, the enhanced couplings between beta and delta/theta might be associated with neuropsychiatric symptoms, whereas the enhanced couplings between alpha and beta might be related to declined memory, cognition and perception in AD patients. In addition, the cross-frequency overcouplings, which reflect enhanced synchronization, might indicate that AD patients need to use more neural resources, especially from non-temporal lobe, to maintain the resting brain state, and the complexity of the neuronal network has been attenuated.

Considering this present study was a preliminary exploration, it had certain limitations. First, likely restricted by the small sample size, we did not observe a marked correlation between the power of cross-frequency coupling and the neurocognitive performance. Second, we failed to use the multiple comparison correction to confirm the statistical significance on each electrode’s level. Therefore, in the future, we need to increase our sample size to confirm the abnormality of EEG oscillations in AD patients.

## Conclusion

In the present study, using wavelet-based EEG analysis, expected for the increased delta and decreased alpha power, an enhanced ongoing gamma rhythm power was found in AD patients. Furthermore, the enhancement of the cross-frequency coupling strength between the beta/gamma and low-frequency bands was observed in AD patients. These current initial observations might provide new evidence for the disturbance of the brain oscillation network in AD and further deepen our understanding of the central mechanisms of AD. Future questions that should be addressed are whether these alterations are related to certain impairments in AD patients and whether the abnormal dynamics of brain oscillations in some core areas affect the global abnormality.

## Author Contributions

JW, YF, XW and HY designed the study and collected the data; JW analyzed the data and wrote the manuscript; and HW and XY conceived the study and supervised the work. All authors contributed to the subsequent drafts and approved the final version.

## Conflict of Interest Statement

The authors declare that the research was conducted in the absence of any commercial or financial relationships that could be construed as a potential conflict of interest.
